# Human Immunodeficiency Virus-Associated Vasculitis: A Case Report of Sensorineural Hearing Loss, Bell's Palsy, and Psoriasis Guttata

**DOI:** 10.7759/cureus.104705

**Published:** 2026-03-05

**Authors:** Beatriz Simão-Parreira, Adriana Costa, Henrique Cerveira, Jorge Almeida, Leila Cardoso

**Affiliations:** 1 Department of Internal Medicine, Unidade Local de Saúde de São João, Porto, PRT; 2 Department of Medicine, Faculdade de Medicina da Universidade do Porto, Porto, PRT

**Keywords:** aortic vasculitis, hiv-associated vasculitis, immune dysregulation, multisystemic inflammation, psoriasis guttate, sensorineural hearing loss

## Abstract

Human immunodeficiency virus (HIV) infection can manifest with diverse and nonspecific symptoms, often delaying diagnosis. Neurological and autoimmune complications have been associated with HIV-induced immune dysregulation. Although HIV-associated vasculitis is rare, its recognition is crucial due to its potential impact on disease progression and management. We report the case of a 62-year-old woman who was initially referred to the autoimmune disease clinic for evaluation of left-sided sensorineural hearing loss (SNHL), which had been fluctuating for two years and showed a response to corticosteroids. One year before the consultation, she developed Bell’s palsy, which was thought to be postviral and resolved with corticosteroid therapy. Over time, she experienced recurrent upper respiratory infections and low-grade fevers. After the first consultation, the patient developed dispersed skin lesions suggestive of psoriasis guttata and cervical and submandibular lymphadenopathy. Laboratory investigations confirmed HIV-1 infection, and the positron-emission tomography computed tomography imaging revealed widespread hypermetabolic lymphadenopathy and active aortic vasculitis, suggesting an inflammatory process related to HIV. Antiretroviral therapy (ART) with dolutegravir/lamivudine was initiated. During early treatment, her psoriasis resolved completely, and there was no recurrence of hearing loss or Bell’s palsy. HIV-associated vasculitis is an infrequent but clinically significant manifestation that can affect vessels of various sizes. The pathogenesis involves direct endothelial damage by HIV, immune complex deposition, and a proinflammatory cytokine cascade. This case suggests that fluctuating SNHL and Bell’s palsy may have resulted from small-vessel vasculitis or direct viral nerve damage, with aortitis further supporting the presence of systemic vascular inflammation. Additionally, psoriasis guttata, emerging during acute HIV infection, was likely driven by immune activation and cytokine dysregulation. The resolution of psoriasis and stabilization of symptoms after ART initiation highlight the central role of immune restoration in mitigating inflammatory manifestations. This case underscores the need for a high index of suspicion for HIV in patients presenting with multisystemic inflammatory conditions. The identification of HIV-associated vasculitis is rare and requires detailed clinical assessment and imaging. The multidisciplinary approach involving otorhinolaryngology, autoimmune disease, and infectious disease was essential for reaching a diagnosis in a timely manner and optimizing patient outcomes. ART remains the cornerstone of treatment, controlling viral replication, reducing immune dysregulation, and improving systemic inflammation. Early diagnosis and prompt initiation of ART play a critical role in preventing complications and ensuring a favorable long-term prognosis.

## Introduction

Human immunodeficiency virus (HIV) infection remains a significant global health concern, with millions affected worldwide [1]. The initial phase of HIV infection typically presents within two to four weeks after exposure, during which many individuals experience acute retroviral syndrome (ARS). This phase is characterized by nonspecific symptoms such as fever, lymphadenopathy, pharyngitis, rash, myalgia, and headache [2]. These manifestations often resemble those of other viral infections, which can lead to misdiagnosis or delayed recognition of HIV infection. It is important to note that while these symptoms are generally self-limited, they are associated with high viral replication and a marked increase in HIV transmission risk. The acute phase is also characterized by a transient decline in CD4+ T-cell counts, which often recovers after the initial immune response [2]. Following the acute phase, individuals may enter a prolonged asymptomatic period, known as clinical latency, which can last several years. There is growing evidence linking HIV infection with exacerbations of autoimmune disorders and vasculitis, which can further obscure diagnosis [3]. Sensorineural hearing loss (SNHL), as seen in this patient, has been associated with both autoimmune conditions and HIV-related vasculitis, while Bell's palsy is also known to occur in the context of HIV infection, often due to viral inflammation of the cranial nerves [4]. Additionally, the development of vasculitis, such as the aortic vasculitis noted in this case, is an important feature of HIV-related vascular inflammation [5]. The interplay between HIV infection, autoimmune conditions, and vasculitis requires heightened clinical suspicion, as it may significantly impact the course and management of patients.

## Case presentation

The patient was a 62-year-old woman, widowed for 14 years, and working as a seamstress. She had no history of smoking, alcohol use, drug consumption, foreign travel, tattoos, or previous transfusions. She had a regular sexual partner for the past two years, with no consistent use of condoms. Her medical history was significant for hypertension treated with lisinopril and hydrochlorothiazide, autoimmune thyroiditis with hypothyroidism treated with levothyroxine, and an anxiety disorder with sertraline. Additionally, she had congenital right-sided SNHL.

She was referred to an autoimmune disease clinic for the evaluation of left-sided fluctuating SNHL that began two years before. The hearing loss responded to corticosteroid therapy but lacked an apparent infectious cause. One year before consultation, she experienced Bell’s palsy, which was attributed to a preceding upper respiratory infection and resolved with corticosteroids. While awaiting an autoimmune assessment, she developed recurrent low-grade fevers, upper respiratory infections, and otitis media with tympanic membrane perforation that resolved without progression to chronic otitis. At her first autoimmune clinic visit, she reported no active autoimmune symptoms, and the physical examination was deemed unremarkable. An initial serologic workup was requested, and a follow-up appointment was scheduled for two months later.

**Figure 1:**
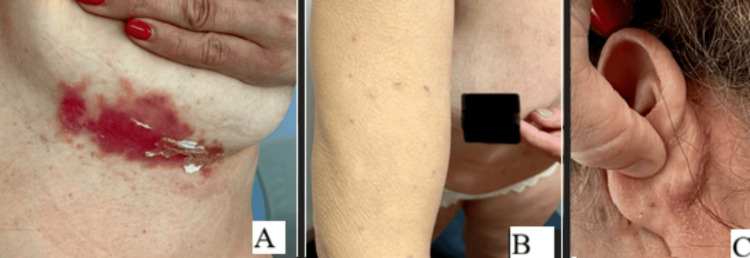
Clinical presentation of patient’s guttate psoriasis affecting different body regions (A) Erythematous, scaly plaques with sharply demarcated borders in the inframammary fold. (B) Multiple small, red, scaly papules scattered over the upper limb and torso. (C) Fine scaling and erythema affecting the retroauricular area

One month later, the patient contacted us after developing nonpruritic, erythematous, scaly skin lesions over the trunk, lower limbs, scalp, and retroauricular areas (Figures 1A-1C), along with new-onset cervical and submandibular lymphadenopathy. For this reason, the patient was scheduled for early observation. On observation, we confirmed the presence of submandibular and cervical lymphadenopathies, and psoriasis guttata was suspected.

The patient had blood drawn for laboratory testing that same day, and a referral was made for biopsy of the skin lesions and lymphadenopathies. We also requested a positron-emission tomography (PET) scan to further clarify the lymphadenopathies.

A few days later, upon consultation of the laboratory test results, the HIV testing returned positive, with a high viral load (128,000 copies/mL) and a CD4 count of 278 cells (ratio CD4/CD8: 0.37) (see Table 1). Following the HIV diagnosis, the patient was urgently referred to an infectious disease specialist and started on dolutegravir/lamivudine. During the early phase of antiretroviral therapy (ART), she developed oral candidosis and vulvar condylomas. Screening for opportunistic infections, including tuberculosis, was negative.

**Table 1:** Complementary study analytical results ESR: erythrocyte sedimentation rate; CRP: C-reactive protein; IgG: immunoglobulin G; ANA: antinuclear antibodies; ANCA: antineutrophil cytoplasmic antibodies; TB: tuberculosis; IGRA: interferon-gamma release assay; CD4 count: cluster of differentiation 4 lymphocyte count

Parameter	Result at admission	Result in December 2024	Reference range
Hemoglobin	12.5 g/dL	-	12.0-16.0 g/dL
Platelets	117 × 10⁹/L	-	150-400 × 10⁹/L
Leukocytes	3.53 × 10⁹/L	-	4.00-11.00 × 10⁹/L
ESR	56 mm/hour	-	0-30 mm/hour
CRP	2.0 mg/L	-	<3.0 mg/L
IgG	2,990 mg/dL	-	600-1,560 mg/dL
HIV-1 RNA (viral load)	128,000 copies/mL	32 copies/mL	20-10,000,000 copies/mL
ANA	Negative	-	-
ANCA	Negative	-	-
Hepatitis B/C	Negative	-	-
Syphilis	Negative	-	-
TB screening (IGRA)	Negative	-	-
CD4 count	-	278 cells/µL	Normal >500 cells/µL

**Figure 2:**
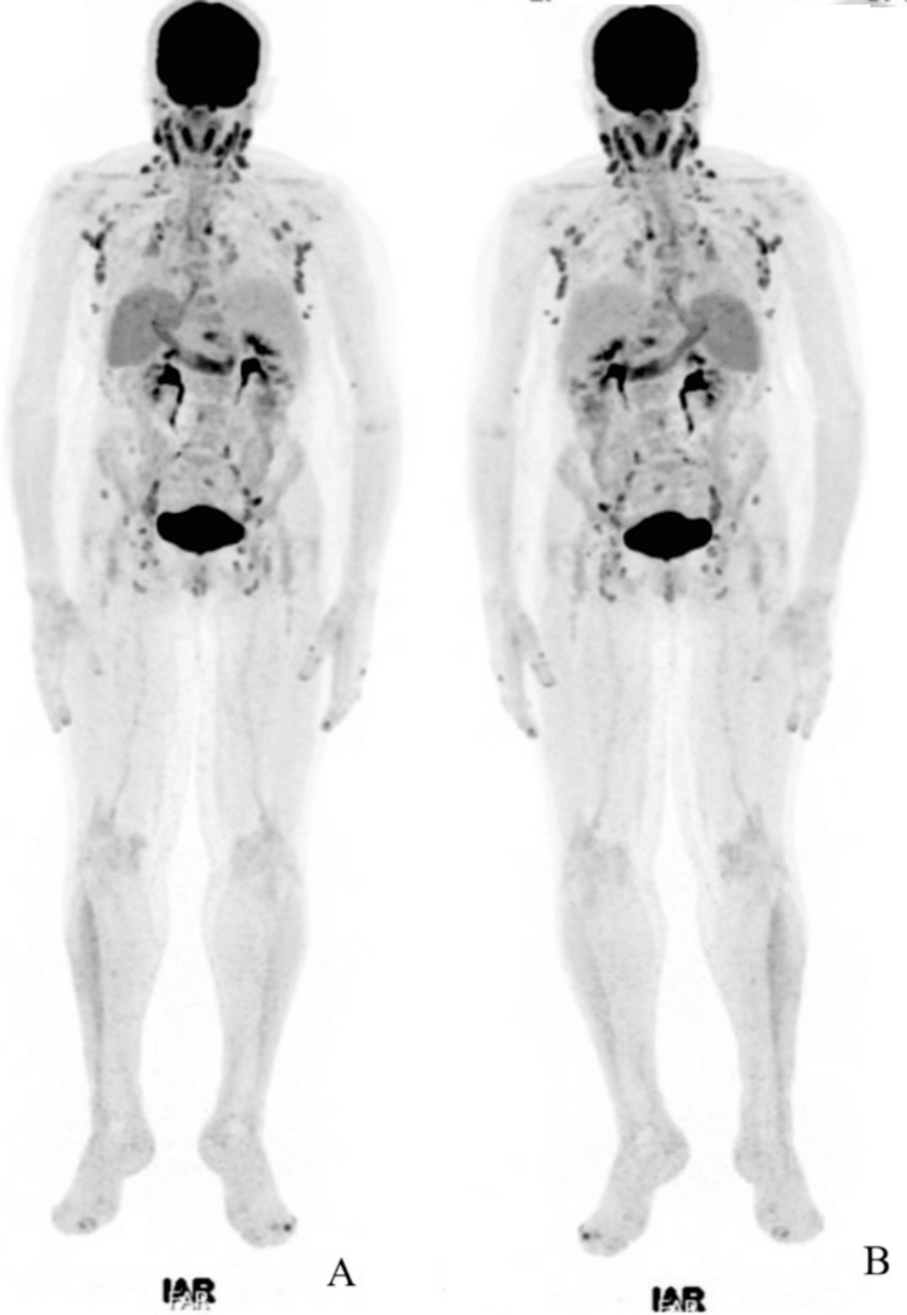
Full body positron-emission tomography scan showing extensive hypermetabolic lymphadenopathies and aortic inflammation (A) Full body scan, anterior view, showing overall lymphadenopathies. (B) Full body scan, posterior view, showing overall lymphadenopathies

**Figure 3:**
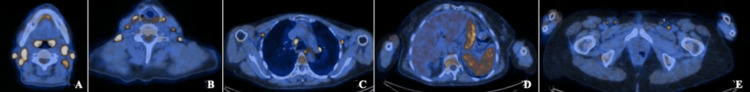
Positron-emission tomography scan cross sections showing extensive hypermetabolic lymphadenopathies and aortic inflammation Cross-section at the mandibular (A) and cervical (B) levels, showing submandibular and cervical lymphadenopathies. (C) Cross section of the thoracic region at the level of the aortic arch, showing paratracheal and axillary lymphadenopathies and inflammatory activity along the ascending aorta, arch, and proximal descending portion. (D) Cross section of the abdominal region, showing homogeneous hyperuptake of [^18^F]FDG at the spleen, suggestive of bone marrow reactivity to an inflammatory condition. (E) Cross section of the pelvic region, showing inguinal lymphadenopathies FDG: fluorodeoxyglucose

The PET computed tomography (CT) imaging revealed extensive hypermetabolic lymphadenopathy, involving the cervical, axillary, mediastinal, intra-abdominal, pelvic, and inguinal regions, raising concerns for either a lymphoproliferative disorder or an inflammatory/infectious process (Figures 2, 3). Additionally, active aortic vasculitis was noted, a finding suggestive of HIV-related vascular inflammation (Figure 3C). The lymph node biopsy showed reactive changes without evidence of malignancy or infection. When the patient was referred for the dermatology consultation, she no longer had any skin lesions, so a biopsy was not performed. Since starting ART, the patient has not experienced any recurrence of psoriasis lesions or hearing loss.

To date, the patient is responding well to the ART regimen, with viral load suppression in the first month of therapy. The psoriasis lesions have regressed, and the patient is due to return for imaging and analytical reevaluation in April 2026.

## Discussion

In Portugal, the HIV epidemic has been notably severe compared to other Western European countries. Historically, the country had one of the highest HIV incidence rates in the region, with 255 new diagnoses per million people in 2002. Since 2014, there has been a 36% reduction in new HIV diagnoses and a 66% decrease in acquired immunodeficiency syndrome (AIDS) cases. In 2023, a total of 876 new HIV diagnoses were reported [6].

HIV infection is a chronic viral disease caused by human immunodeficiency virus, characterized by progressive immune system depletion, leading to increased susceptibility to opportunistic infections and, if untreated, the development of AIDS [2]. Data on the exact number of HIV cases diagnosed during the primary infection phase in Portugal and Europe are limited. Early detection during this phase is challenging due to the nonspecific nature of symptoms and the potential for misdiagnosis. Efforts to promote early testing and awareness are crucial to identify and manage HIV infections promptly.

In the context of HIV infection, there is an increased risk of immune dysregulation and exacerbation of autoimmune conditions. While specific studies for autoinflammatory states in HIV infected patients are limited, it is known that HIV-related inflammation can increase the risk of autoimmune diseases such as vasculitis, psoriasis, and other inflammatory disorders [3].

HIV-associated vasculitis is relatively uncommon (reported incidence of 1%-5% of advanced HIV infection) and can affect various vessel sizes and types. Literature indicates that HIV-associated vasculitis predominantly affects small- and medium-caliber vessels and is more frequently seen in individuals with high viral load or very low CD4 counts. The pathophysiology involves direct viral effects on the vascular endothelium, immune complex deposition, and the dysregulated immune response characteristic of HIV infection. Clinically, HIV-associated vasculitis can present with diverse manifestations, depending on the specific vessels involved and the extent of the inflammatory process [5,7].

In the context of primary HIV infection, fluctuating SNHL and Bell’s palsy may be associated with small-vessel vasculitis, direct nerve damage due to the rapid replication of the HIV virus during this phase, or reactivation of other latent viruses, such as herpes simplex or varicella zoster. In the presented case, we thought that we were probably dealing either with direct damage to the facial and auditory nerves by the HIV replicating virus or with HIV associated small-vessel vasculitis. In light of the finding of aortitis on the PET scan and considering the symptomatic improvement of the hearing loss and the resolution of Bell’s palsy after corticosteroid treatment with a tapering regimen, it is most likely that this is a case of small-vessel vasculitis induced by the autoinflammatory response associated with primary HIV infection [4,8-10].

Finally, the patient presented with a new onset of psoriasis guttata that resolved after initiation of ART. Psoriasis is a chronic autoimmune skin condition characterized by hyperproliferation of keratinocytes and inflammatory infiltration. The onset of psoriasis in this patient is likely attributed to the proinflammatory state associated with primary HIV infection. During ARS, there is a surge in immune activation marked by high levels of proinflammatory cytokines such as tumor necrosis factor-α, interleukin-6, and interferon-γ, which play a critical role in both the pathogenesis of psoriasis and the immune response to HIV. This hyperinflammatory environment can trigger or exacerbate autoimmune conditions, including psoriasis, by promoting keratinocyte proliferation and sustained T-cell activation. Additionally, the dysregulation of the immune system in early HIV infection, characterized by an imbalance between pro- and anti-inflammatory mediators, creates a milieu conducive to autoimmune skin reactions. The resolution of psoriasis following the initiation of ART further supports the hypothesis that immune dysregulation in primary HIV infection was the key driver of the skin eruption, as ART restores immune homeostasis and reduces systemic inflammation [11,12].

The treatment of HIV-associated vasculitis requires a dual approach: controlling the underlying HIV infection with ART and managing the inflammatory vascular process [5]. Initiation of ART is the cornerstone of therapy, as it helps suppress viral replication, restore immune function, and reduce systemic inflammation, which can lead to the resolution of vasculitic manifestations in many cases. In patients with severe or symptomatic vasculitis, adjunctive immunosuppressive therapy, such as corticosteroids, may be required to control acute inflammation and prevent end-organ damage. However, the use of immunosuppressive agents must be carefully balanced, as excessive immune suppression can increase the risk of opportunistic infections, particularly in individuals with low CD4 counts. In refractory cases or those involving large-vessel vasculitis, additional immunomodulatory therapies, such as methotrexate, azathioprine, or cyclophosphamide, may be considered under close monitoring [5]. Given the potential complications associated with both HIV infection and vasculitis, a multidisciplinary approach involving infectious disease, autoimmune disease, and vascular specialists is essential to ensure optimal management and long-term patient outcomes.

The follow-up of HIV-associated vasculitis in this patient requires close clinical and imaging surveillance to monitor disease progression and response to ART. For small-vessel vasculitis, such as the suspected involvement in the patient's fluctuating SNHL and Bell’s palsy, prognosis is generally favorable with ART initiation, as immune reconstitution tends to resolve vasculitic activity over time. However, recurrent episodes of hearing loss or cranial nerve involvement warrant periodic audiometric testing and neurological evaluation. If symptoms persist or worsen, additional immunosuppressive therapy, such as corticosteroids, may be considered, though with caution due to the risk of opportunistic infections. For large-vessel vasculitis, particularly aortic vasculitis detected on PET-CT, long-term monitoring is essential due to the risk of vascular complications, including aneurysm formation, vessel stenosis, or ischemic events [13]. Serial imaging with PET-CT or MRI angiography at regular intervals is recommended to assess disease activity and detect structural changes in the aorta. In many cases, ART alone can lead to regression of vascular inflammation, but in patients with persistent vasculitis, corticosteroids or other immunosuppressants may be required. The prognosis of large-vessel vasculitis in HIV remains variable, depending on the degree of vascular involvement and response to therapy. While controlled HIV infection generally leads to better outcomes, untreated or severe cases can result in life-threatening vascular complications.

Given the patient’s favorable response to ART, with no recurrence of psoriasis or hearing loss, and the absence of new systemic symptoms, her long-term prognosis appears promising. However, continued follow-up with infectious disease specialists, rheumatologists, and vascular specialists remains crucial to prevent complications, ensure immune recovery, and detect any signs of vascular progression.

## Conclusions

This case underscores the complex interplay between HIV infection, immune dysregulation, and inflammatory manifestations. Our comprehensive diagnostic approach led to the recognition of HIV-associated vasculitis, a rare but significant complication. The otorhinolaryngologist’s suspicion of an immune-mediated etiology for the patient’s fluctuating hearing loss was pivotal in guiding further investigation. PET-CT imaging confirmed active aortic vasculitis, supporting an HIV-driven autoimmune process. The rapid improvement following ART initiation highlights the role of immune activation in these manifestations. This case reinforces the need to consider HIV in patients with unexplained multisystemic symptoms and the value of interdisciplinary collaboration in managing its complications.
